# The Ayeyarwady River (Myanmar): Washload transport and its global role among rivers in the Anthropocene

**DOI:** 10.1371/journal.pone.0251156

**Published:** 2021-05-13

**Authors:** Edgardo M. Latrubesse, Edward Park, Karl Kästner

**Affiliations:** 1 Graduate Program in Environmental Sciences-CIAMB, Universidade Federal de Goiás, Goiânia, Brazil; 2 National Institute of Education and Asian School of the Environment, Nanyang Technological University, Singapore; 3 Institute of Environmental Sciences, Brandenburg University of Technology, Cottbus, Germany; Duy Tan University, VIET NAM

## Abstract

The Ayeyarwady (Irrawaddy) is the second largest river of Southeast Asia and one of the rivers with the highest load of suspended sediment delivered to the sea in the world. The Ayeyarwady is the lifeline of Myanmar which concentrates the majority of the population and GDP of the country. It is the main way of transport, a source of fluvial aggregates for development projects, hydropower, and the basin plays a major role in food supply and irrigation. Despite the Ayeyarwady ranking amongst the world’s largest rivers and its vital importance to Myanmar, scarce research has been undertaken to understand its morphodynamics and sediment transport regime. Current load estimates still heavily rely on the only systematic study of sediment transport dating back to the 19th century. Here, we provide a novel estimate for the recent washload sediment transport based on a field calibrated remote sensing model of surface suspended sediments concentrations. We show that the Ayeyarwady has likely become the river with the second or third largest delivery of washload to the sea in the world since it has so far been much less affected by damming compared to the vast majority of other rivers.

## Introduction

The Ayeyarwady (Irrawaddy) River is one of the less known large rivers in the world and the second-largest river of Southeast Asia in water discharge, after the Mekong [1]. It is still having a natural hydrological regime because regulation by dams is incipient. The system is also a hotspot of biodiversity, but the basin is very vulnerable because of human environmental pressure such as mining, dredging, deforestation, and proposed dams are increasing rampantly [2, 3]. The Ayeyarwady Delta is also considered the least impacted of the large deltas of Asia, and the most extremely prone to floods by tropical storms and coastal storm surges. Myanmar is also among the 15 nations in the world most severely affected by floods [4, 5]. It is known to suffer the most infamous and deadly floods by the interactions between tropical storms-cyclones and fluvial floods such as tropical storm Nargis in 2008, which produced 138,000 fatalities.

There are also geopolitical and socio-economic factors that make the Ayeyarwady a priority for environmental research. It is the main artery of the country and concentrates most of the population and GDP of Myanmar. For all these factors, the Ayeyarwady is considered a top priority among the world’s transboundary river basins for risks related to hydro-political tension and the lack of water governance at a national level.

The largest rivers of the world are suffering increasing environmental pressure that demands multiple national and internationally coordinated actions [6, 7]. A major concern has been the dramatic decrease of the sediment fluxes of large rivers to their floodplains, deltas, and the Ocean. The role of sediments for “healthy” rivers has been underestimated by years despite the claims of its fundamental importance to sustain channel migration rates, sustainable ecological channel-floodplain connectivity, and its vital role to preserve the functionality and services provided to humans by the deltas [8, 9].

On a global scale, Southeast Asia has been considered the region with the largest sediment yields [10]. However, because of a scarcity of studies, the suspended sediment fluxes of the Ayeyarwady are poorly understood, and only a few reports and publications have been generated. The literature on the sediment fluxes from the 1960s to the early 2000s had been based on results by Gordon [11]. Robinson et al. [12] provided new estimations based on Gordon and a limited set of new field data, and Furuichi et al. [13] reassessed Robinson’s results. Recently, Baronas et al. [14] estimated the load based on water samples combined with ADCP measurements in the wet and dry season. However, no systematic field surveys to collect hydro-sedimentological data have been carried out in modern times.

Estimations of suspended sediment transport in large alluvial rivers include wash load (silt and clay) and suspended bed material (sand). However, a large proportion of the national and state agencies or data available in the literature, only publish values of “suspended” load without discriminating the grain sizes distribution and the proportions of sand in suspension and mud [15], among many others.

In large rivers, concentrations of the individual size fractions can strongly vary through the water column, and the role of sand in suspension can be particularly relevant or even dominant. Thus, the calculation of sandy load (bedload and suspended sand) is usually approached in combination through a variety of methods that range from hydraulic transport equations to acoustic doppler profilers (ADCPs) applications and field data [16] and many others. However, complications arise when suspended sediment sampling is conducted with an integrated sampler through the water column, as it can include sand in suspension and washload.

An additional factor is that the largest rivers of the world are located in tropical countries with a limited historical record of hydro-sedimentological data [1]. While some large rivers such as the Mississippi in the USA have been monitored for decades, and the grain sizes of the suspended sediments are systematically determined and reported by the United States Geological Survey (USGS), in most countries, and particularly those in the tropics, where many of the largest rivers are located, the governmental hydrological agencies rarely systematically monitor sediment transport. Furthermore, even when available, the scarce data on suspended sediments concentrations do not discriminate grain size. Moreover, often, in many countries, access to governmental hydro-sedimentological databases is difficult and the availability of hydrological data is restricted to the public.

To tackle the problem, it is necessary to apply methods that discriminate the different modes of transport. A challenge with monitoring sand transport is that whatever technique is applied it usually is limited to a number of episodic measurements of sand concentration and discharge that are correlated into a sediment load rating curve. This considerably increases the uncertainty in the predicted transport. However, calculations of wash load transport can be more effectively tackled as it is characterized by a more uniformly distributed concentration of the muddy and silty sediment through the water column. Furthermore, field calibrated remote sensing models of Suspended Sediment Surface Concentration (*SSSC*) are state-of-the-art tools that allow multi-temporal measurements of sediment concentration close to the water surface in large rivers that are dominated by washload transport. Thus, these models have been used to determine continuous washload fluxes of large rivers (e.g. [17–20]).

Here, we present a new field calibrated model of *SSSC* for the Ayeyarwady River and a new estimation of washload transport patterns at Pyay, by estimating over 16 years of surface suspended sediment concentration (*SSSC*) since 2000 at an 8-day temporal resolution. The goal of this study is twofold. First, to gain a better understanding of the sediment load of the Ayeyarwady River through remote sensing, as there is neither a sediment monitoring programme in place nor easy access for researchers to field sites. Secondly, to determine how the load of the Ayeyarwady River compares to other large rivers exposed to considerable anthropogenic influence.

## The Ayeyarwady River at Pyay

**Fig 1:**
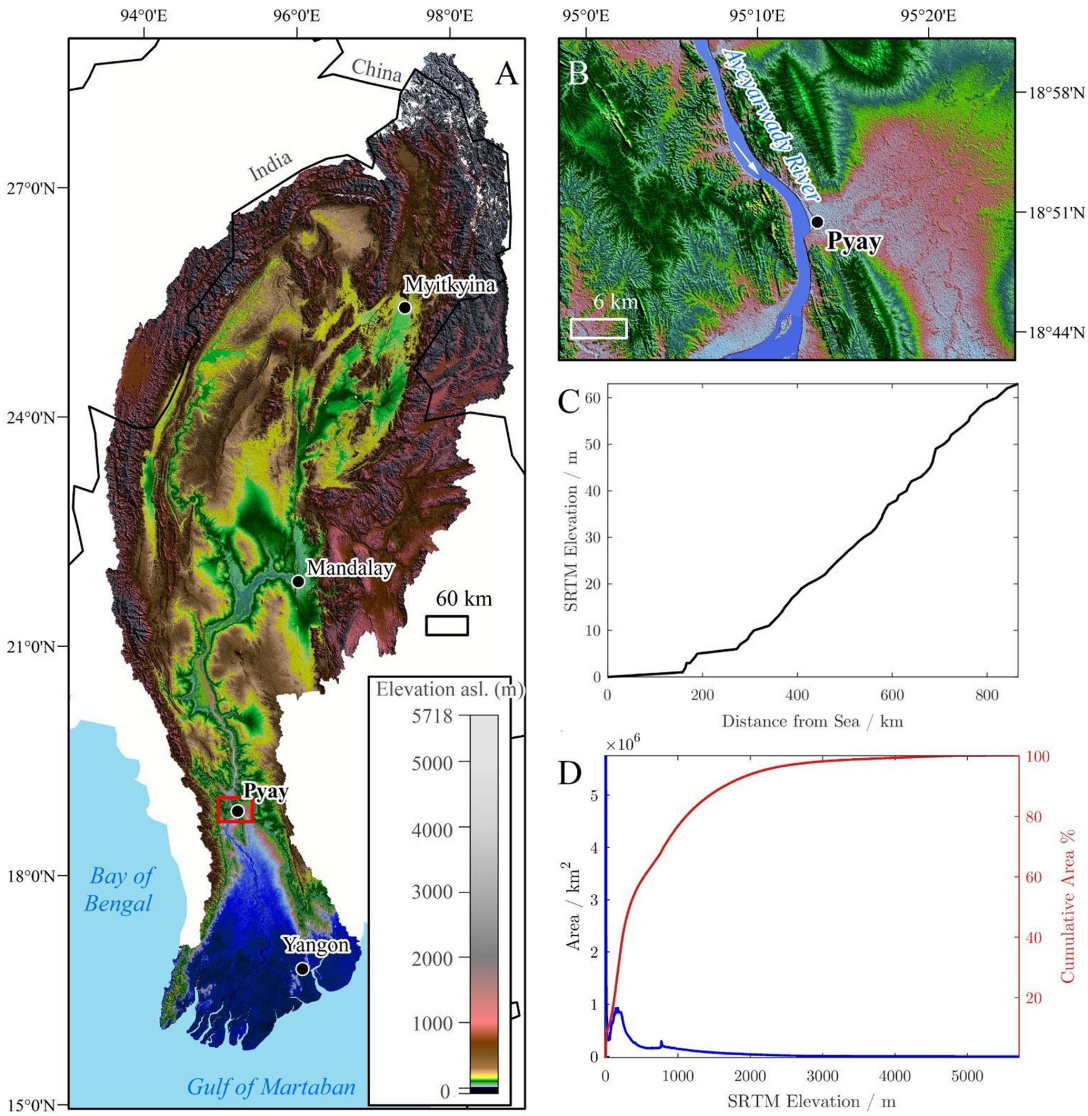
a) Topographic map of the Ayeyarwady basin (hillshade SRTM DEM). b) Map of Pyay station where washload flux is estimated. c) SRTM elevation of the river surface and d) the basin hypsometry. SRTM was downloaded from USGS EROS (eros.usgs.gov).

The Ayeyarwady River basin extends over an area of 421,000 km^2^ reaching from the Himalayas to the Gulf of Martaban. Although the maximum elevation exceeds 5,700 m, the mean elevation of the basin is 656 m. 17% are lowlands (Fig 1). From Mandalay to the Andaman Sea, the river flows along an 860 km long reach. This part of the river can be subdivided into four major reaches with slopes ranging from 7 to 4.7 cm/km alternating narrow, geological constrained reach and broader alluvial reaches.

We calculate the sediment flux of the Ayeyarwady at Pyay, located 120 km upstream of the delta. It is a suitable location for monitoring discharge and sediment fluxes, as the banks of the river are constrained at this site, and the Department of Meteorology and Hydrology of Myanmar (DHM) operates a gauge station. No significant tributaries join the river downstream of Pyay, and the measurements are representative (also the largest) of the total discharge and sediment fluxes of the Ayeyarwady before bifurcating into a distributary pattern in the delta. Pyay is located 50 km upstream of the historical measurement site of Gordon [11].

At Pyay, the Ayeyarwady is on average 1,100 m wide. The hydrological regime is typically monsoonal with floods during the northern hemisphere summer (June-September) and a marked dry season and lower flows in winter (January-March). The mean annual discharge from 2000 to 2016 was 365 km^3^ y^-1^ (11,600 m^3^ s^-1^). The discharge reaches an average wet-season maximum of 3.9 10^4^ m^3^ s^-1^. Because of the high seasonality, the mean monthly discharge in August is on average 10 times higher than the discharge in February.

## Materials and methods

We built a field-calibrated remote sensing model in the virtual gauge station to quantify surface suspended sediment concentration (SSSC). We used Moderate Resolution Imaging Spectroradiometer (MODIS) 8-day composite data from both Terra (MOD09Q1) and Aqua (MYD09Q1) satellites at 250 m resolution. The 8-day window is sufficient to derive a continuous time series, as the sediment concentration and surface reflectance of large rivers do not change substantially over this time span. Images were calibrated using field-measured *SSSC* data collected at Pyay in July 2017. Daily discharge at Pyay for 1966 to 2020 was compiled from reports [21] and the current website of the Department of Meteorology and Hydrology (DMH).

### Surface water samples and surface suspended sediment concentration (SSSC)

We followed a similar protocol to HYBAM, Mertes et al. [22] and Park and Latrubesse [18] to collect surface water samples and to process suspended concentration data. A total of 36 Surface water samples were collected using 500 ml bottles along the river on July 11^th^ and 12^th^, 2017. At this time the discharge was 16,000 m^3^ s^-1^, corresponding to mean flow conditions. Cellulose acetate membranes (0.45 μm) are used to filter sediments (Merck Millipore) and weighted after drying 24 hours to retrieve SSSC.

### Particle size of surface suspended sediments

**Fig 2:**
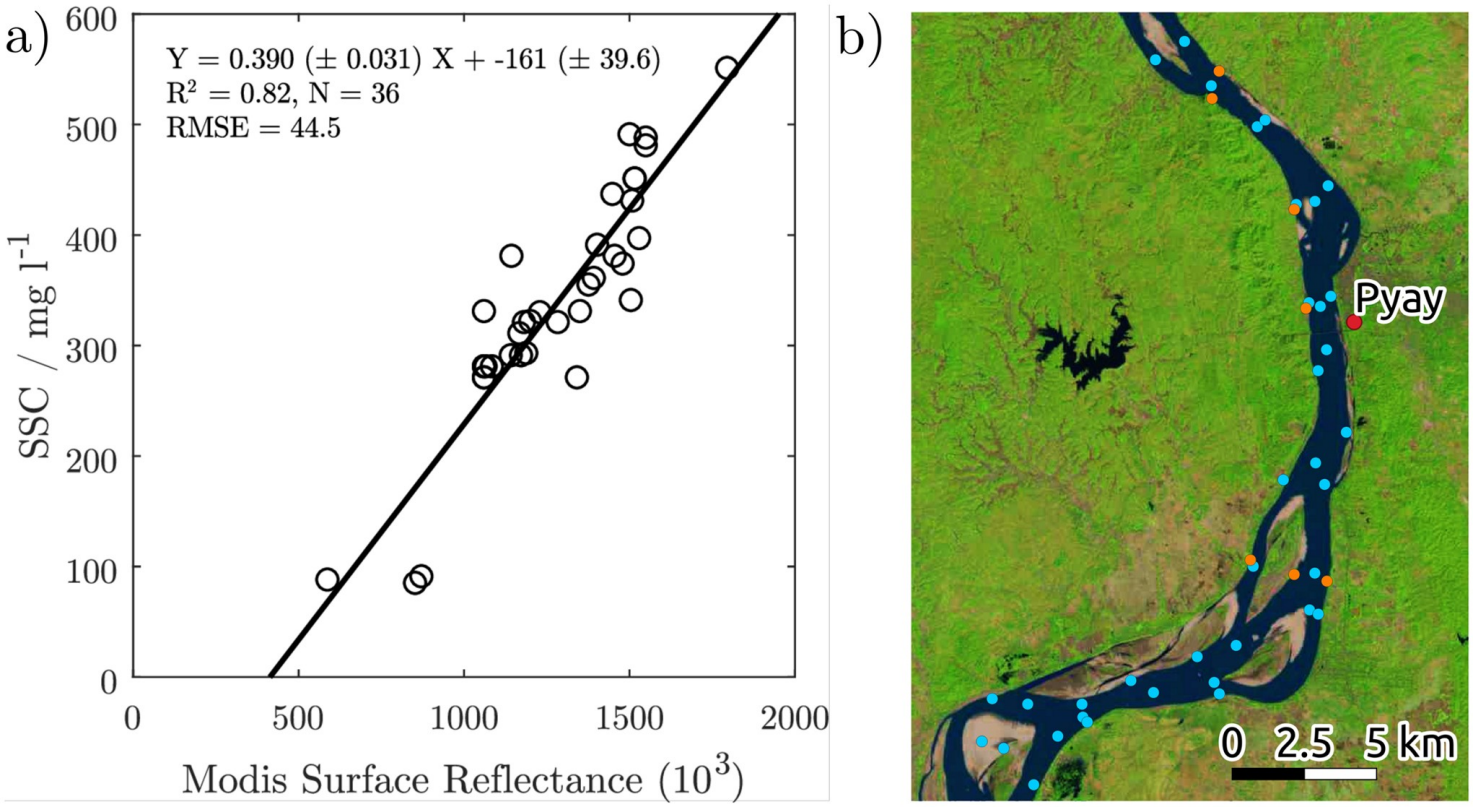
a) Fit of the sampled surface suspended sediment concentration (*SSSC*) to MODIS surface reflectance. b) Location of sampling sites near Pyay. Cyan points indicate surface water samples and orange points indicate bed material grab samples. Background Landsat 8 image was downloaded from USGS EROS (eros.usgs.gov).

Six of the surface water samples were collected in 20 litres containers for particle-size analysis. The recovered sediment was treated with 30% hydrogen peroxide (H_2_O_2_) and 10% hydrochloric acid (HCl) (Fig 2). The grain-size distributions were measured using a Malvern Mastersizer 3000 Hydro EV laser grain-sizer. The distributions of the different particle sizes were quantified with Grandistat [23].

### Remote sensing pre-processing and calibration

Satellite images were corrected for the effects of atmospheric gases, aerosols, and cirrus clouds [24] which then has been generated from the daily images using the constrained-view angle maximum value composite method (L3-V5) [25]. Surface reflectance from Band1 (red band centered around 645 nm) was used to estimate surface sediment concentrations because reflectance between 600 and 800 nm shows very high and constant sensitivity to *SSSC* in turbid inland waters Mertes et al. [22]. The high radiometric sensitivity of MODIS bands is also well-suited to detect subtle changes in water-leaving radiances as typical reflectance of sediments at the surface water is concentrated in a low-end portion of the whole reflectance range. MODIS data were downloaded from the Land Processes Distributed Active Archive Center (LP DAAC, https://lpdaac.usgs.gov/) both for the analysis period 2000–2015 as well as the calibration year 2017 (image date July 12^th^ 2017 h27v07 in the MODIS tiling system). Collected images are re-projected to the Universal Transverse Mercator Projection zone 47N using the MODIS reprojection tool (available at https://lpdaac.usgs.gov/) and then were resampled to 250 m using the bilinear interpolation. Further calibration processes including controls on pixel quality, solar zenith angle, and interpolation of unqualified pixels followed methods described in Park and Latrubesse [18]. Subsequently, we fitted SSSC against the surface reflectance by linear regression.

Our Method is based on the principle that the surface reflectance, as captured by the satellite sensor, can be correlated with the concentration of sediments in the water body [26–28]. However, sand transport cannot be determined from surface reflectance in the presence of near-surface sediment. It is because, firstly, the near-surface concentration of fine sediment is much larger than that of the sand, and secondly, the optical reflectance of fine sediment per unit mass far exceeds that of the sand. Remote sensing models for estimating *SSSC* of rivers are site-specific and usually are not applied universally. It is because surface reflectance values are not only determined by the *SSSC* but also related to the grain size distribution and inherent optical properties of the sediment [19]. Thus, to generate a representative and reliable result, it is necessary to build a statistically robust regional model for each river or even each river-reach. For example, in the Brazilian Amazon, it is necessary to calibrate site-specific remote sensing models for different locations, as the correlations between *SSSC* and surface reflectance significantly differ from each other [19]. For the Ayeyarwady, we use 36 *SSSC* samples collected at Pyay to calibrate the remote sensing model. The 36 water samples were analysed for suspended sediment concentration and six of them for grain size.

## Results

The calibrated model predicts the SSSC from the surface reflectance reliably with an R^2^ of 0.82 (Fig 2a), i.e. the variability of *SSSC* is efficiently explained by the level of surface reflectance. The fit is statistically significant at a 95% confidence level. The overall root mean square error (RMSE) is 44.5 mg/l. Given that the range of *SSSC* around Pyay can be 1,500 mg/l (around 500 to close to 2,000 mg/l, Fig 2a), the calculated RMSE represents less than 3% of the *SSSC* range. The residuals are distributed evenly throughout the entire range of SSSC, supporting the application of a linear fit without systematic error.

**Fig 3:**
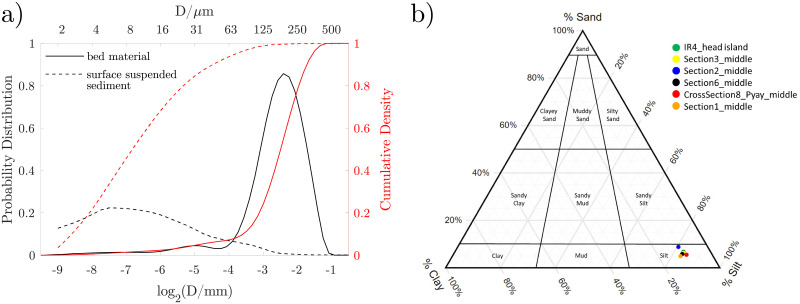
a) Average grain size distribution of the bed material (solid) and average particle size distribution of the surface suspended sediment (dashed) at Pyay. b) Classification of the surface suspended sediment. Sample coordinates and size distributions of individual samples are provided in the S1 File.

Surface sediments consist mostly of medium silt with a D50 of 8 um. The distribution is skewed towards coarser grains with 91 to 95% consisting of mud and 5% to 9% very fine sand. The surface concentration of sand possibly increases during high flow (Fig 3). The disjoint distributions of SSSC and bed-material support that the SSSC concentration indeed corresponds predominantly to washload and can therefore be considered to be independent of the suspended bed-material load.

**Fig 4:**
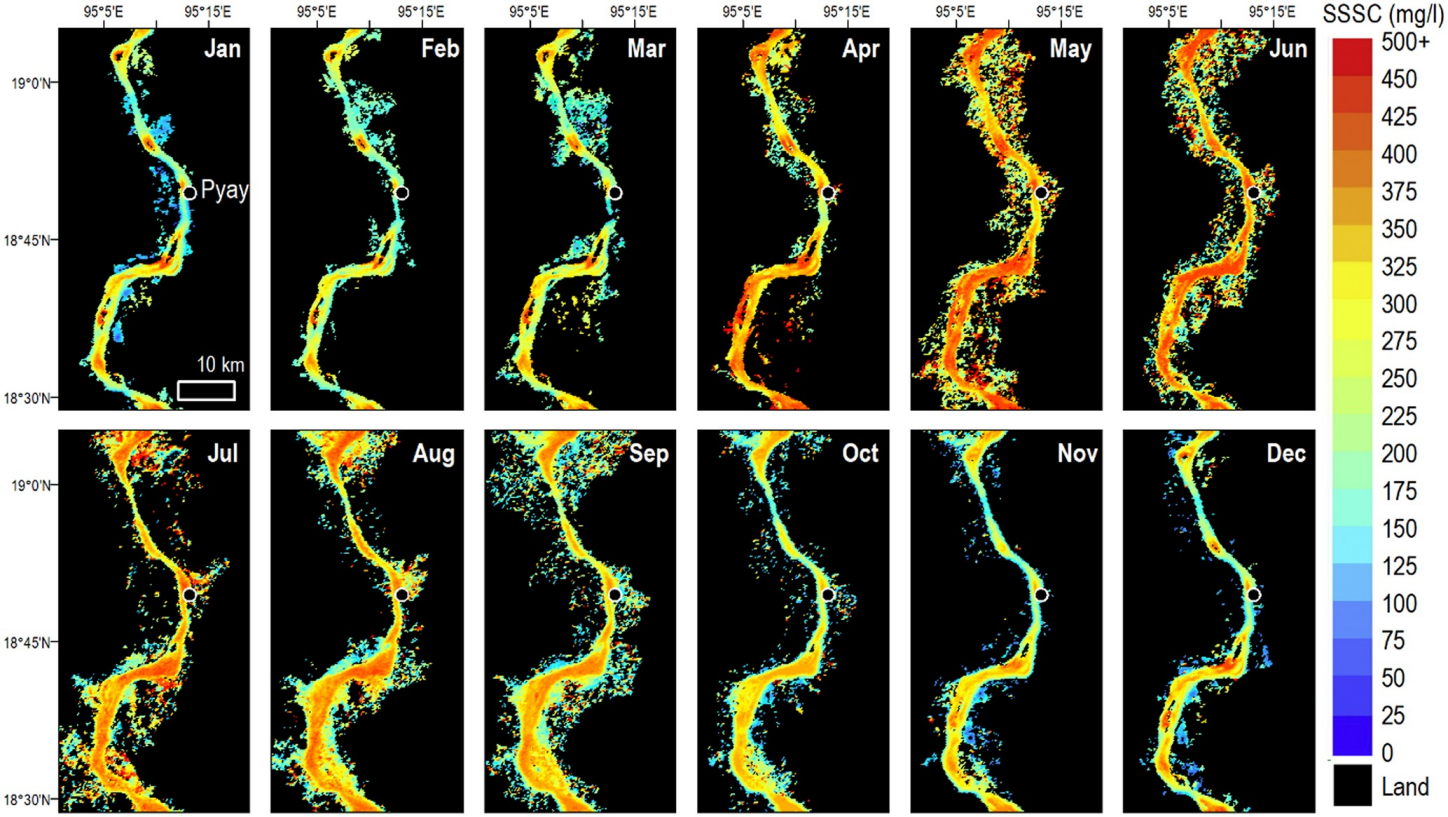
Monthly SSSC of the Ayeyarwady at Pyay, average over the years 2000–2015. Derived from 60 MODIS satellite images per month on average.

The method reliably resolves the spatiotemporal dynamics of SSSC at Pyay, similar to previous applications in other large rivers [7, 9, 26, 29]. In particular, the seasonal variation of the SSSC due to the monsoonal cycle, with high values during the wet season and low values during the dry season is well resolved (Fig 4).

**Fig 5:**
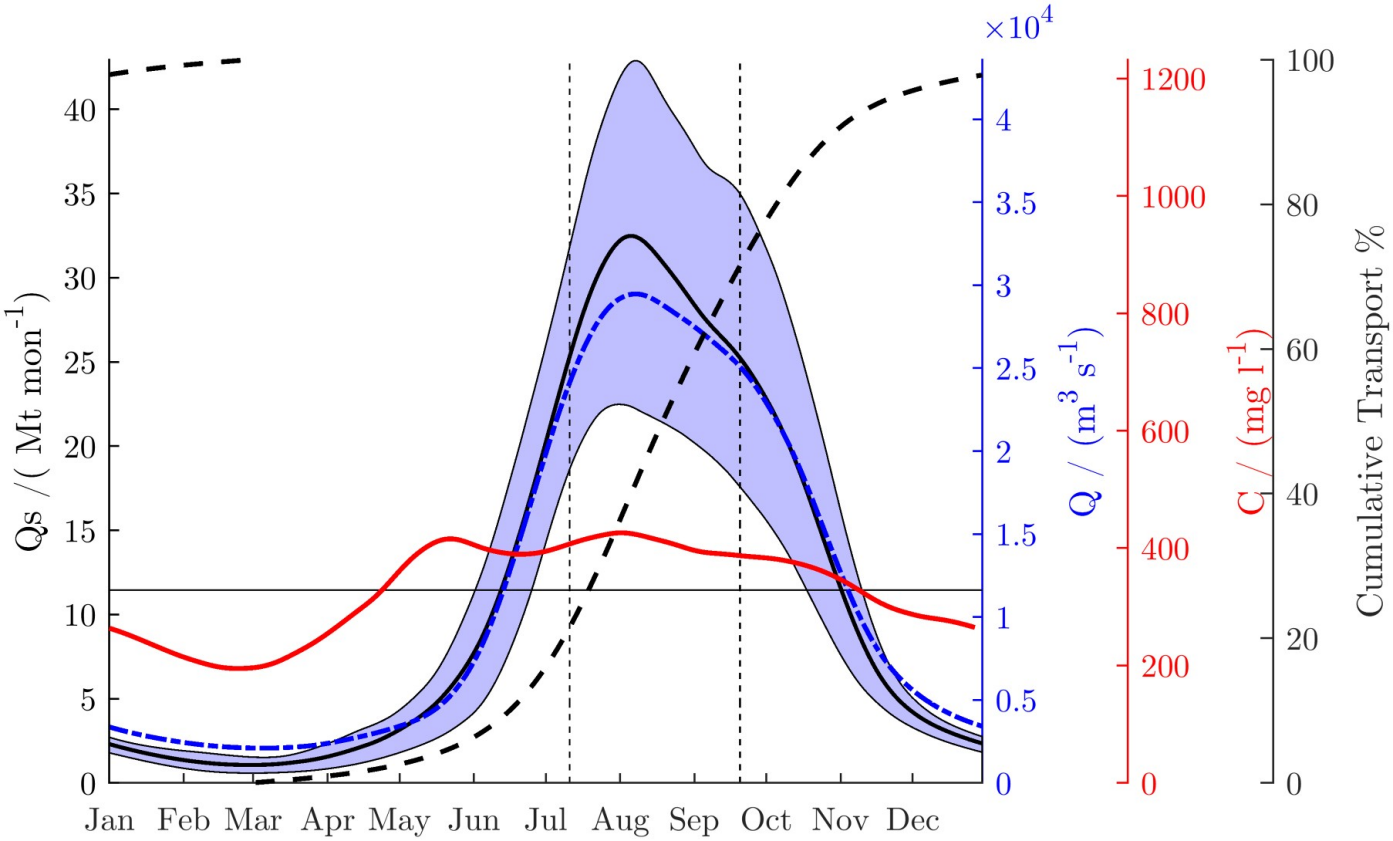
Wash load transport (black solid), concentration (red solid) and discharge (blue dash-dotted) of the Ayeyarwady River at Pyay between 2000 and 2015. Bold solid lines indicate average. Shaded area indicates intervals between 16% and 84% percentiles. Time series have been smoothed over a 30-day interval. 50% of the annual wash load is transported in less than three months during the wet season (vertical dashed line). All axes scaled proportionally to the annual mean (horizontal line).

The monsoonal variation of the sediment concentration is almost in phase with the discharge, but the *SSSC* increases slightly earlier than the discharge during the rising stage (Fig 5).

**Fig 6:**
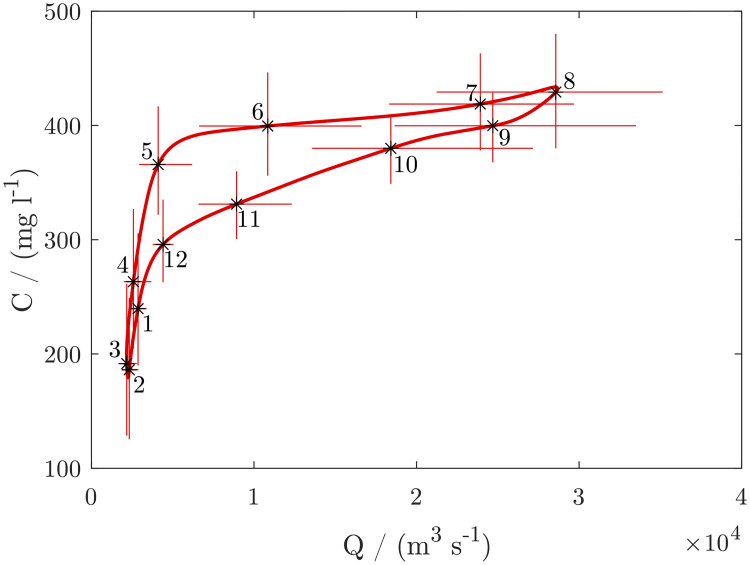
Hysteresis between the discharge and washload concentration over the annual cycle, with a higher sediment concentration during the rising and a lower sediment concentration during the falling hydrograph. The red line indicates the concentration averaged over the 16 years and smoothed over a 30-day interval. Crosshairs indicate 16^th^-84^th^ percentile ranges for discharge and concentration on the 15^th^ day of each month.

**Fig 7:**
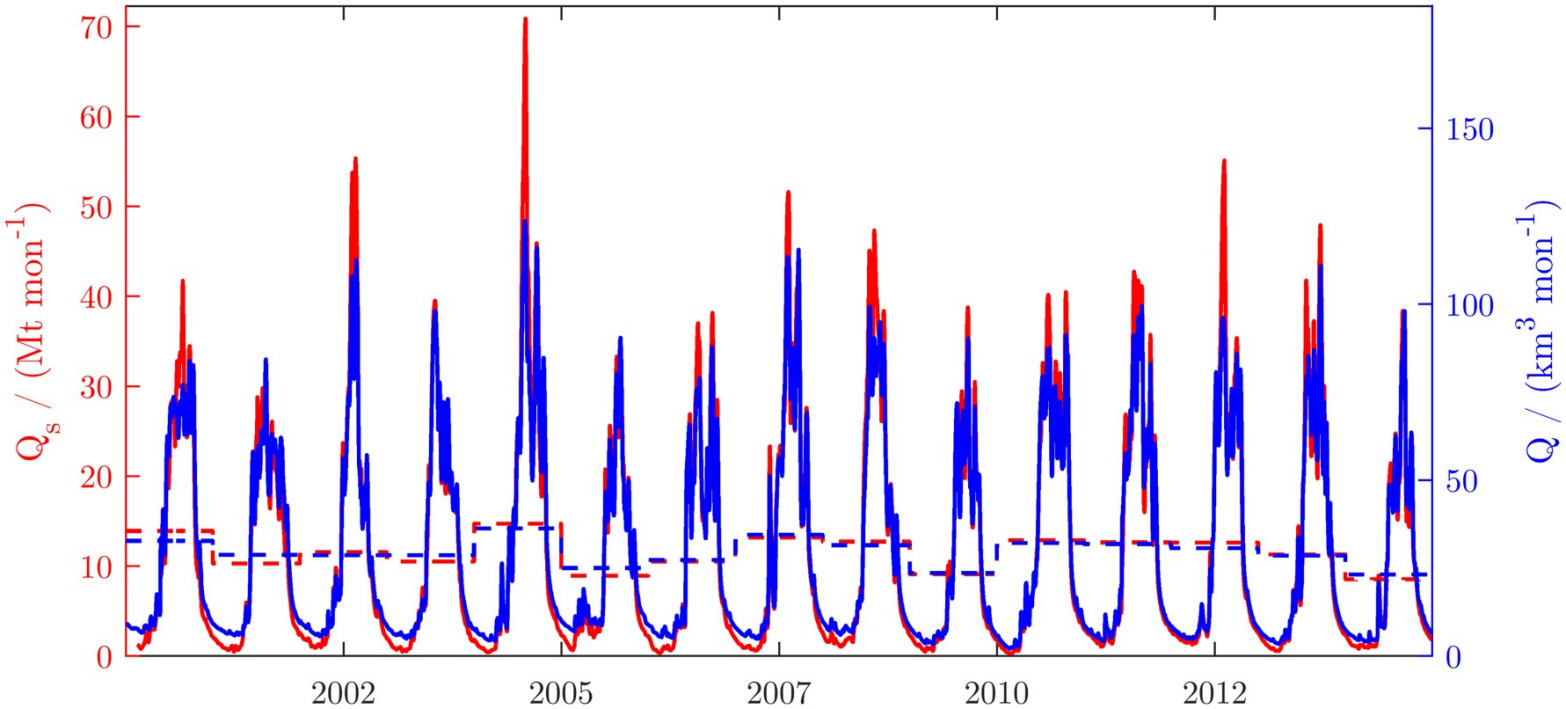
Daily discharge and wash load transport of the Ayeyarwady at Pyay. Dashed lines indicate the annual averages. The load was estimated by multiplying the gauged discharge with the weekly estimate of SSSC interpolated to days.

Thus, *SSSC* reaches a peak in July while the floods reach a peak in August. During the fall of the hydrograph, the *SSSC* is more in phase. There is thus a hysteresis between discharge and SSSC over the seasonal cycle (Fig 5). On average, the *SSSC* ranges from 200 to 450 mg/l (Fig 6). The hysteresis is also evident in the times series (Fig 7), which shows that during most years, the sediment concentration of the first discharge waves is higher than that of the following waves.

Sediment concentration and washload transport strongly decrease during the dry season, because of the low discharges in the Ayeyarwady mainstem and the decrease of discharge, erosion and sediment transport in its tributaries. On average, 50% of the sediment is transported during less than three months from mid-July to mid-September. Between 2000 and 2015, the mean annual wash load transport was 144 Mt yr^-1^, of which we estimate about 10% was suspended sand (Fig 7).

**Fig 8:**
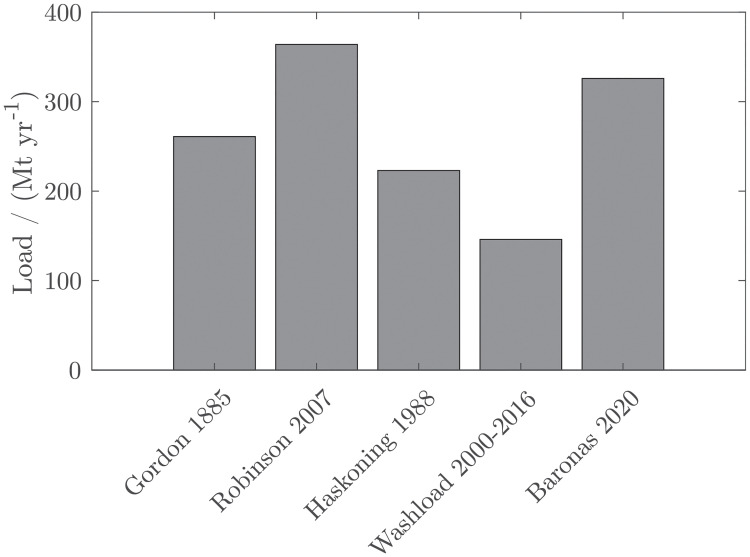
Comparison of washload with historic load estimates.

The total suspended load, i.e. the combined wash load and suspended bed material load, of the Ayeyarwady has been estimated by Robinson et al. [12] to be 364 Mt yr^-1^. Robinson et al. [12] corrected historic estimates by Gordon (261 Mt yr^-1^) for systematic underestimation of fine sediment. We estimate the wash load to be 144 Mt yr^-1^. Baronas et al. [14] estimated the total load to be 326 Mt/y, based on the systematic difference between his measurements and prediction with Robinson’s rating curve. The difference of 10% is however much smaller than the uncertainty of both estimates (~25%). Based on a total load of 364 Mt yr^-1^, the suspended sediment thus consists of 40% washload and 60% suspended bed material. This result is supported by the sediment rating curve developed by Haskoning in 1988 [21]. This curve gives a load of 278 Mt/yr for the calibration period of Haskoning (1968–1974) and 223 Mt/yr predicted from discharge between 1968 and 2020, the latter being 62% of the total load estimated by Robinson Fig 8.

### Relation between surficial suspended sediment and discharge

**Fig 9:**
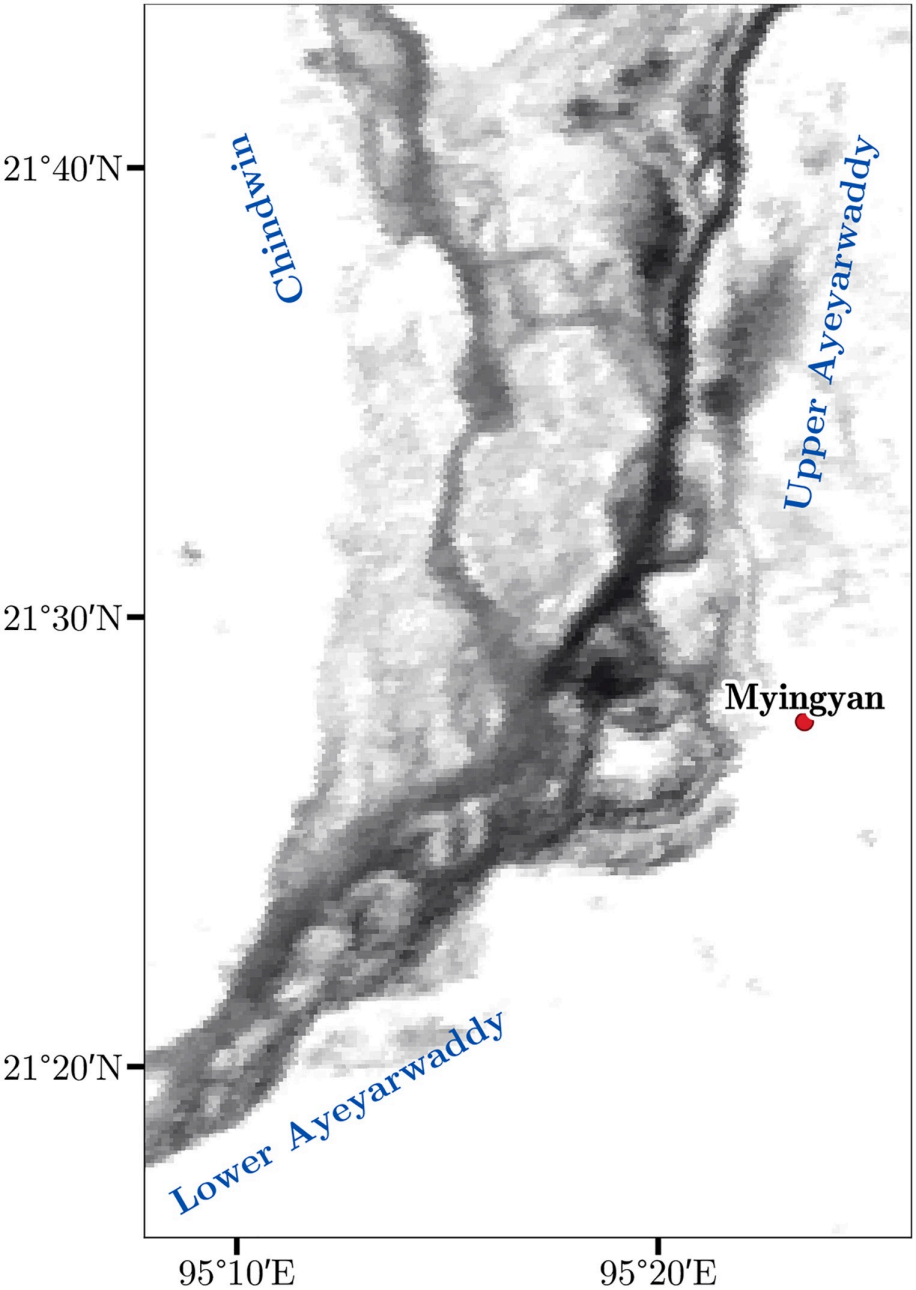
Prediction of the sediment concentration from the river discharge. Averages for the observed years smoothed over a 30-day interval.

In a first attempt, we fit a simple sediment rating curve SSSC = a Q^b^. We fit the coefficients with the method of non-linear least squares to avoid the bias towards low flows affecting the fits in log-space. With SSSC in g/l and Q in m^3^/s, the resulting coefficients were a = 0.0460 and b = 0.2184. The rating curve has a relatively poor fit with a coefficient of determination (R^2^) of 0.56. This because SSSC leads the discharge during the onset of the wet season and lags behind the discharge during the end of the wet season Fig 9. There is thus an excess of sediment at the beginning and end of the wet season. The hysteresis is very pronounced, as the concentration on average leads the annual hydrograph by over two months. This cannot only be explained by in-channel processes, as the time span is considerably longer than the passage of a flood wave along the entire river. Hysteresis in rivers associated with in-channel processes have typically a much shorter time span on the order of days [30, 31]. We hypothesize that the excess of sediment at the beginning of the wet season is caused by the washing of fine sediment into the river by overland flow, and the excess of fine sediment during the receding hydrograph is caused by a gradual winnowing of the fine particles from the bed, as in the absence of rainfall the river is fed by ground-water only, which does not replenish the fine sediment.

We fitted several more complex rating curves that accounted for non-stationarity and seasonal depletion effects, which did not considerably improve the fit. The best model we found was the combination of the sediment rating curve with a strong forward shift and a strong low-pass filter: SSSC = a Q_f(t+T_s_)^b^. Here T_s_ is the shift in time and Q_f(t) = c Q_f(t-Δt) + (1-c) Q(t) is the low-pass filtered discharge with Δt the time step of 1 day and c = exp(-Δt/T_l_) the filter coefficient. The fitted coefficients are a = 6.44 10^−4^, b = 0.4558, T_l_ = 80.8 d, T_s_ = -72.3 d. This model slightly improves the fit to R^2^ = 0.67. The overall fit is still poor as the strong-low pass filter removes the peaks of individual flood waves and reproduce mostly the seasonal dynamics. This is because the hysteresis of the concentration with respect to the annual hydrograph does not carry over to individual flood waves. Concentration and discharge can peak nearly simultaneously. Visual matching of concentration and discharge peaks was ambiguous due to the strong seasonal hysteresis. A cross-correlation analysis of the filtered discharge and concentration time series, where seasonal variations with a wavelength longer than one month were removed, did not reveal a significant hysteresis for individual flood waves. As a comparison, a simple model that predicts the concentration for each day by the average annual sedigraph independently of the discharge has an R^2^ of 0.75.

It is astonishing, that the strong hysteresis of the Ayeyarwady has so far caught little attention. Even if one accounts for that the total suspended sediment concentration will be stronger linked to the hydrograph than the surficial suspended sediment concentration, as the former also comprises of suspended bed material. Only Robinson [12] has noticed the different hysteresis for the rising and falling limb similar to us. Neither Furuichi [13] nor Haskoning [21] did notice the hysteresis as they relied on precomputed concentration provided by the DHI agency in Myanmar which apparently did not account for the hysteresis. This stresses the importance of direct access to time series of measured sediment concentrations.

### Origin of the suspended surficial sediment

**Fig 10:**
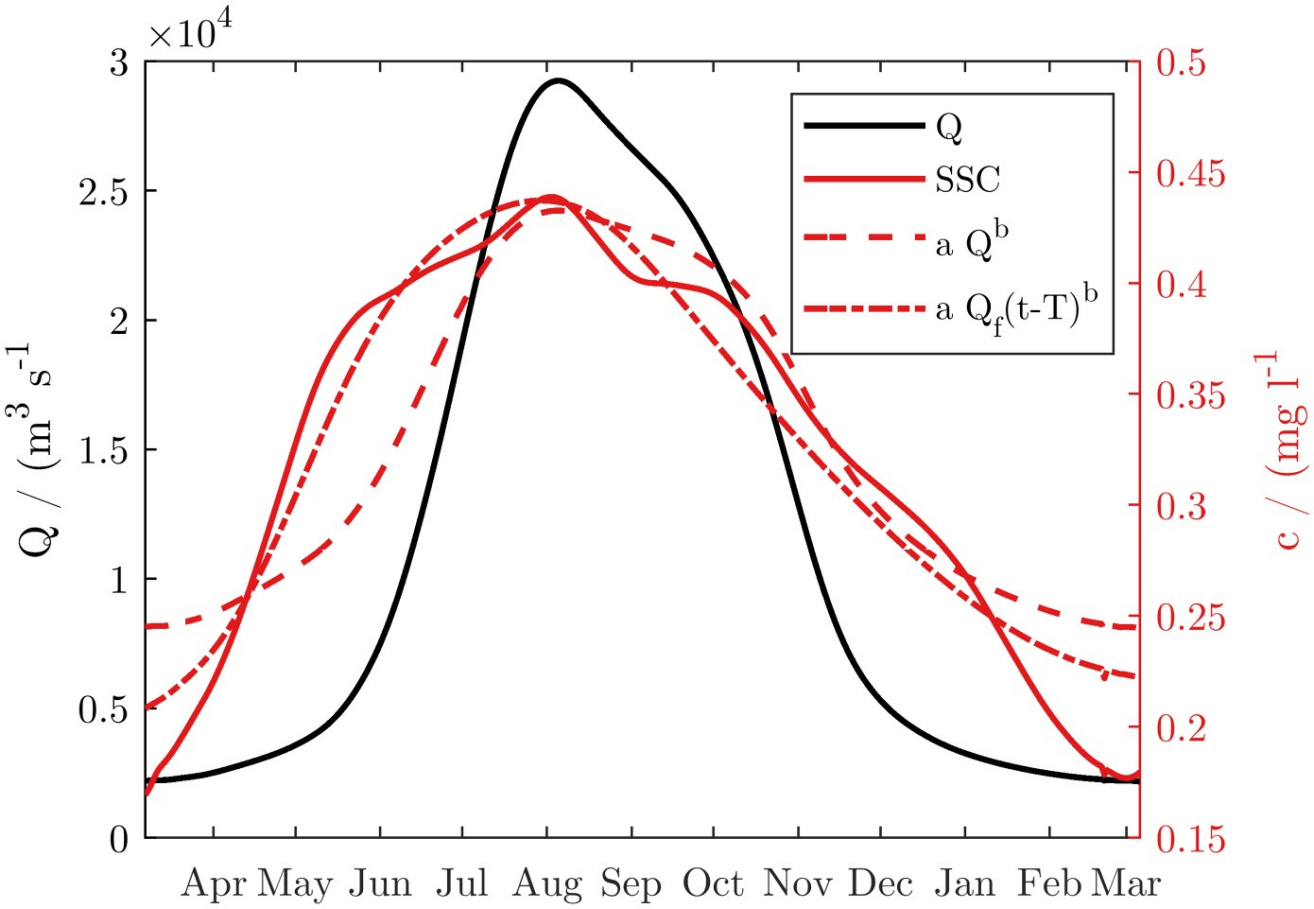
Surface reflectance at the Chindwin confluence for July averaged over the years 2002–2020 (MODIS Terra and Aqua). Higher reflectance of the Chindwin and Lower Ayeyarwady indicate that the bulk of SSC is derived from the Chindwin basin, and not the Upper Ayeyarwady.

The provenance of sediment of the Ayeyarwady was studied on the hand of chemical analysis of the bed-material by Garzanti et al. (2016) [32]. They showed that the majority of the sediment of the Ayeyarwady stems from the Upper Ayeyarwady and Chindwin basins. The rivers meet near Myingyan 360 km upstream of Pyay. Despite the Chindwins basins smaller area of 115 000 km^2^ compared to 200 000 km^2^ of the Upper Aryeyawady, they showed that the Chindwin contributes with 200 Mt y^-1^ more than half of the total load of the Aryeyawady, which was estimated in the same study in the range 350–400 Mt y^-1^. Garzanti et al. (2016) [32] attributed this to higher erosion rates of 1700 t km^-2^ y^-1^ in the Chindwin basin to only 1000 t km^-2^ y^-1^ in the Upper Ayeyarwady. The Chindwin receives its largest sediment through erosion of turbite rocks in the Indo-Burmese range, while sources the Upper-Ayeyarwady consist mostly of harder to weather gneiss and plutonic rocks. However, they analyzed bed material and their inference is less certain for SSC than for bed-material transport. Surface reflectance observed by the MODIS satellites allows to independently test Garzanti et al. (2016)’s [32] hypothesis. At the confluence, the reflectance of the Chindwin river is considerably higher than of the Upper Ayeyarwady (Fig 10). Downstream of the confluence the reflectance of the Lower Ayeyarwady is closer to that of the Chindwin rather than to that of the Upper Ayeyarwady. While a budget estimate for both sub-basins will require another field calibration of both rivers upstream of the bifurcation, the surface reflectance supports the hypothesis that the Chindwin contributes the larger share of the SSSC.

## Large rivers and washload transport in the Anthropocene: The role of the Ayeyarwady

**Fig 11:**
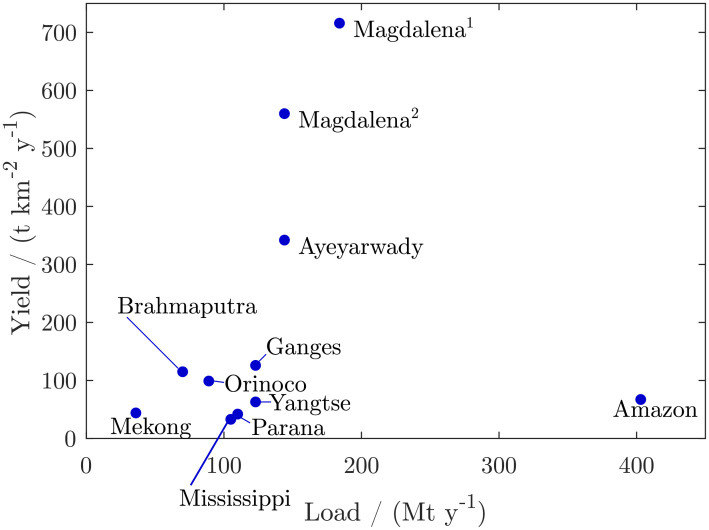
Current annual washload and corrected values of washload as subtracted from the total suspended load of the world’s largest rivers delivering suspended sediments to the Oceans (data and explanations in Table 1).

Since the XX century, humans have been intensely modifying and regulating river systems. Among other multiple negative consequences, the sediment fluxes of rivers to the Ocean decrease more and more [6, 8, 33–35]. When considering rivers that were partially or non-regulated in the last century, we can see that the Ayeyarwady used to rank as the fifth-largest suspended load system in the world [12]. Our perception is that until decades ago, although the imprecise estimations of suspended load, the Ayeyarwady was, with high probability the sixth in the world in suspended sediment transport, after the Amazon, Yangtze, Brahmaputra, Ganges, and Mississippi [15, 34]. Based on those computations, the Ayeyarwady used to be during the XX century the fourth largest in the world in sediment yield among the largest rivers discharging to the Ocean, after the Brahmaputra, Magdalena, and Ganges (Fig 11 and Table 1).

**Table 1:** Current annual washload of the world’s largest rivers.

River	Dis- charge	Area	Wash-load	Yield	Reference
	m^3^ s^-1^	10^6^ km^2^	Mt y^-1^	t km^-2^ y^-1^	
Amazon	209000	6000	403	67.2	Washload and discharge by using Park and Latrubesse (2014), (2019) at Obidos
Yangtse	28380	1943	123	63.3	total suspended load from Guo et al. (2019)
Ganges	10900	980	123	125.5	corrected value of washload as subtracted from total suspended load by Rahman et al., 2018, and using Garzanti et al. (2011), and Roy and Sinha 2017 as a reference for suspended sand. Mean annual discharge from Darby et al. (2015) [58].
Brahmaputra	21900	610	70	114.8	corrected value of washload as subtracted from total suspended load by Rahman et al., (2018), using as a reference Garzanti et al. (2011). Mean annual discharge from Darby et al. (2015)
Mississippi	18000	3200	105	32.8	washload estimated from Nittrouer et al. (2008), Knox and Latrubesse, (2016); Joshi and Xu, (2015)
Ayeyarwady	13600	421	144	342.0	Washload and mean annual discharge, this study
Mekong	12683	810	36	44.4	Total suspended load and mean annual discharge from Thi Ha et al. (2018) and suspended sand from Hackney et al. (2020).
Orinoco	33320	900	89	98.9	Total suspended load and mean annual discharge, Gallay et al. (2019) and Yepez et al. (2018) [59].
Magdalena (1)	7390	257	184	716.0	Total suspended load and mean annual discharge Restrepo et al. (2016), Restrepo et al. (2017).
Magdalena (2)	7390	257	144	560.3	Total suspended load and mean annual discharge Restrepo et al. (2016), Restrepo et al. (2017)
Parana	18000	2600	110	42.3	Washload and mean annual discharge, Amsler et al. (2007) and Amsler and Drago (2009)

However, the impacts on sediment fluxes by dams and other human activities around the world have been drastically increasing [6, 8]. The decrease of sediment load also provokes a remarkable reduction of the effective sediment yield in the basins, because, despite some areas still contributing a significant amount of sediments to the rivers, a large proportion of the sediment is currently trapped inland by dams and other human interventions. Thus, here, we compute the *effective sediment yield* as the sediment yield related to the current sediment fluxes reaching the Oceans for the total area of the catchment. Specific comparisons on wash load transport in large rivers were not yet conducted. Our new estimations allow us to compare the Ayeyarwady with the other tenth-largest rivers of the world in washload discharge, that can reach the Ocean. Many of them are mega rivers [36], and except the Mississippi, all are related to the tropical regions, orogenic belts, and monsoonal regimes [1]. They are the Amazon, Yangtze, Orinoco, Parana, Mississippi, Brahmaputra, Ganges, Mekong, and Magdalena.

The Ganges and Brahmaputra are two major basins that merge close to the Gulf of Bengal, and usually, they are considered as a single input to the Ocean. Although it is correct, it is better to consider both river systems individually as they drain different regions and flow as a single system only for a short distance before reaching the Ocean. New estimations for the Ganges and Brahmaputra show that sediment transport is declining for both systems. Historically, it was nearly 1,000 Mt yr^-1^ for both combined systems draining to the Gulf of Bengal. The present values are likely only half as large, and a continued decreasing trend of 10 Mt per year has been estimated [37]. The contemporary sediment flux in the Ganges-Brahmaputra system is approximated (for 2015) as around 500 Mt yr^-1^ with around 220 Mt yr^-1^ for the Ganges and 250 Mt yr^-1^ of suspended sediments for the Brahmaputra [37]. However, those studies do not discriminate the washload from the suspended sand load. Previous punctual studies point to an averaged proportion of 40 to 70% of suspended sand of the total suspended sediment load for the Ganges and Brahmaputra, respectively [38] and in the Ganges, the most detailed studies of effective discharge discriminating suspended sand are for Kanpur or upstream [39, 40], and cannot be directly interpolated to the lowermost reaches. Suspended sand, upstream of Kanpur, typically represents ~20% of the total suspended load [40]. By cautiously incorporating those averages and uncertainties, it can be assumed that the Ganges and Brahmaputra perhaps are transporting in average, something like 123 Mt yr^-1^ to 75 Mt yr^-1^ of wash load (mud) while in contrast, the remaining proportion of the suspended load is sand in suspension. Those estimations are only rough averages as the Ganges and Brahmaputra are characterized for an irregular monsoonal regime of sediment transport [37] and the monsoons months (June-September) are responsible for >80% of the annual of the suspended sediments and represent 60% of the annual total discharge [39].

The Yangtze also experienced an enormous decrease in annual sediment load due to human impacts such as dams [41, 42]. Currently, its total suspended sediment load is estimated to have been decreased to merely 123 ± 20.5 Mt yr^-1^ [43]. However, these results do not discriminate between washload and sand in suspension as separated populations of the total suspended load.

The present sediment supply of the Mekong to the sea has also abruptly decreased from a historical average of ~145–160 Mt/yr before 2003, to merely 40 ± 20 Mt yr^-1^ by 2016 [44]. The decline in sediment load is the consequence of the extensive construction of dams during the last two decades and secondarily land use/land cover changes (LULC) and riverbed mining activities in the basin [45–47]. However, these data also do not discriminate in the budget the proportion of washload and suspended sand. If computing the estimates of 6 ±2 Mt yr^-1^ of suspended sand by Hackney et al. [48], it can implicate that the mean annual wash load is currently close to ~34 Mt yr^-1^.

The Mississippi River is another system that experienced intense engineering and land use and land cover changes since the end of the XIX century and particularly during the XX century. Currently, the Mississippi River transports not more than ~105–128 Mt yr^-1^ of suspended load at Tarbert Landing, the gauge station upstream of the diversion with the Atchafalaya River [49]. Downstream, at Saint Francisville, it reaches an average of ~92 Mt yr^-1^ and decreases to 67 Mt yr^-1^ at Baton Rouge. 12.8 to 35% of the total suspended load consists of sand, as it varies with the flow stage [50, 51]. It points to approximated values below 110 Mt yr^-1^ of wash load at Tarbert Landing before the river enters the lowermost reach and delta.

The Parana has been strongly impacted by dams in the headwaters of the basin in Brazil, but the major source of washload is Andean tributaries that are still not regulated. Consequently, there are still large fluxes of washload toward the delta and La Plata estuary, reaching 110 Mt yr^-1^ [52].

The Orinoco River is another of the top ten mega rivers in water discharge [36]. Based on recent results obtained by using a similar methodology to the one we used for the Ayeyarwady calculations, the average washload was estimated in ~89 Mt yr^-1^ [26].

Into the current environmental context of disruption and degradation faced for the large rivers of the world, the Ayeyarwady, together with a few other large rivers such as the Amazon, and the Magdalena, ranges among the largest rivers of the world carrying yet substantial amounts of washload to the Ocean in relatively natural, i.e., not highly regulated by dams, conditions.

The Magdalena River is a relevant case. It drains the northern Andes of Colombia, and the mainstem is not regulated by dams. However, contrary to the rivers discussed above, the Magdalena River is experiencing an increase in suspended load and sediment yield [53, 54]. Rapid LULC since 1980 resulted in deforestation of 87% of the basin area by 2010, and an increase of 33% of suspended sediment load from 2000 to 2010 [55]. Other authors have pointed to a reduction of suspended sediment transport from 2000 to 2010 in the lowermost reach [56]. It was estimated that during pristine pre-human conditions, the Magdalena River had a sediment load ranging from 34 to 84 Mt yr^-1.^ Until recently, the estimated total suspended load was 144 Mt yr^-1^ [31, 53], but new results indicate a recent increase in the last few years to 184 Mt yr^-1^ [55]. The suspended sediment load of the Magdalena is dominantly silt, but estimations from the proportion of sand and washload in the fluvial reaches upstream the delta are not available. Results in the delta region suggest ~7.1% of suspended sand during the high-discharge season to ~1.9% during the low discharge season [57].

## Conclusion

Our comparative analysis of the washload transport of the largest rivers of the world demonstrates that the Ayeyarwady most likely ranks, after the Amazon, as the second or third largest river of the world in terms of washload, in close to that of the Rio Magdalena, and also as the third or second one in effective washload-sediment yield. The uncertainty in ranking the Ayeyarwady third or seconds is because the Magdalena total annual suspended sediment fluxes seem to be larger, but in both systems, the grain size of the suspended sediment proportions and the proportions of suspended sand to washload deserves more detailed analyses.

Regarding the Ayeyarwady, further challenges are faced to quantity the sandy suspended sediment fluxes in some precise way, because it is a difficult task, particularly in large monsoonal rivers.

As Southeast Asia’s last surviving river of an environmental “rivercide” (environmental destruction) that has devastated the natural functioning of the rivers across the world at an alarming rate, particularly since the second half of the XX century, the Ayeyarwady gains a global relevance, deserving urgent attention by Myanmar authorities for searching sustainable alternatives for the management of the basin, but also the consideration of the global society, scientists, decision-makers and stakeholders as one of the last fluvial wonders of nature that can flow relatively freely to the world’s Oceans.

## Supporting information

S1 File (DOCX)
