# Supplementary material for: The Ayeyarwady River (Myanmar): Washload transport and its global role among rivers in the Anthropocene
Source: PLoS One. 2021 May 13;16(5):e0251156. doi: 10.1371/journal.pone.0251156 (PMC8118563; doi:10.1371/journal.pone.0251156)
Supplement: S1 File — (DOCX) [file pone.0251156.s001.docx]

S1 File.

Supplementary information for “THE AYEYARWADY RIVER (MYANMAR): WAHSLOAD TRANSPORT AND ITS GLOBAL ROLE AMONG RIVERS IN THE ANTHROPOCENE”, Latrubesse et al. 2020

Content:

1) Echo-Sounder Cross-Sections and Bottom-Samples

2) Location and sediment concentration of surface samples

3) Particle size of suspended sediment near surface

4) Grain size of bed material

**1) Echo-Sounder Cross-Sections and Bottom-Samples**

| **Cross-Section** | **Date** | **Coordinate** | **Sample Time** | **Sample Coordinate** |
| --- | --- | --- | --- | --- |
| 1 | 11/07/2017 | 18^0^ 53’23.7” 95^0^ 10’32.7” 18^0^ 52’53.00”95^0^ 10’23.9” | 12:06 | 95.174007 18.886568 |
| 2 | 11/07/2017 | 18^0^ 50’47.1” 95^0^ 11’59.6” |  | 95.207175 18.847822 |
| 3 | 12/07/2017 | 18^0^ 43’48.5” 95^0^ 12’32.8” |  | 95.208231 18.816765 |
| 6 (branch) | 12/07/2017 | 18^0^ 44’13.4” 95^0^ 11’02.6” | 14:04 | 95.185616 18.736559 |
| 8 | 12/07/2017 | 18^0^ 48’55.7” 95^0^ 12’12.0” |  | 95.204086 18.731809 |
| IR4 Island Head | 12/07/2017 | 18^0^ 43’56.5” 95^0^ 11’54.1” |  | 95.202206 18.756246 |

**2) Location and sediment concentration of surface samples**

| **Sample** | **Latitude** | **Longitude** | **SSSC / (mg l^-1^)** |
| --- | --- | --- | --- |
| 0 | 18.87242 | 95.18805 | 354 |
| 1 | 18.68137 | 95.09512 | 396 |
| 2 | 18.67912 | 95.10225 | 373 |
| 3 | 18.66764 | 95.11199 | 329 |
| 4 | 18.77659 | 95.21609 | 321 |
| 5 | 18.72122 | 95.20326 | 292 |
| 6 | 18.71987 | 95.20612 | 480 |
| 7 | 18.70704 | 95.16618 | 490 |
| 8 | 18.69533 | 95.17332 | 450 |
| 9 | 18.69893 | 95.17165 | 430 |
| 10 | 18.69983 | 95.14432 | 380 |
| 11 | 18.69601 | 95.15169 | 330 |
| 12 | 18.68858 | 95.1284 | 310 |
| 13 | 18.687 | 95.12982 | 280 |
| 14 | 18.69263 | 95.12816 | 270 |
| 15 | 18.68272 | 95.12007 | 280 |
| 16 | 18.69285 | 95.11033 | 290 |
| 17 | 18.69466 | 95.09869 | 330 |
| 18 | 18.7309 | 95.18377 | 290 |
| 19 | 18.71041 | 95.17902 | 280 |
| 20 | 18.7327 | 95.20516 | 260 |
| 21 | 18.76196 | 95.19518 | 320 |
| 22 | 18.76038 | 95.20873 | 90 |
| 23 | 18.76714 | 95.20588 | 87 |
| 24 | 18.79594 | 95.20706 | 84 |
| 25 | 18.80246 | 95.20992 | 380 |
| 26 | 18.83194 | 95.21467 | 550 |
| 27 | 18.83239 | 95.21015 | 390 |
| 28 | 18.83194 | 95.20326 | 320 |
| 29 | 18.85488 | 95.20992 | 270 |
| 30 | 18.856 | 95.20255 | 320 |
| 31 | 18.85735 | 95.19685 | 320 |
| 32 | 18.87445 | 95.19067 | 280 |
| 33 | 18.88524 | 95.16642 | 270 |
| 34 | 18.90458 | 95.14741 | 450 |
| 35 | 18.91065 | 95.15929 | 436 |

**3) Particle size of suspended sediment near surface**

|  | **SAMPLE STATISTICS** |  |  |  |  |  |  |
| --- | --- | --- | --- | --- | --- | --- | --- |
|  |  | **IR4_head island** | **Section3_middle** | **Section2_middle** | **Section6_middle** | **CrossSection8_Pyay_middle** | **Section1_middle** |
|  | ANALYST AND DATE: | EOS, 7/28/2020 9:41:52 AM | EOS, 7/28/2020 9:54:50 AM | EOS, 7/28/2020 10:13:18 AM | EOS, 7/28/2020 10:29:19 AM | EOS, 7/28/2020 10:52:06 AM | EOS, 7/28/2020 11:05:11 AM |
|  | SAMPLE TYPE: | Bimodal, Poorly Sorted | Unimodal, Poorly Sorted | Bimodal, Poorly Sorted | Bimodal, Poorly Sorted | Bimodal, Poorly Sorted | Bimodal, Poorly Sorted |
|  | TEXTURAL GROUP: | Mud | Mud | Mud | Mud | Mud | Mud |
|  | SEDIMENT NAME: | Fine Silt | Fine Silt | Fine Silt | Fine Silt | Fine Silt | Fine Silt |
| METHOD OF | MEAN | 18.01 | 20.90 | 19.16 | 16.58 | 17.59 | 15.82 |
| MOMENTS | SORTING | 22.93 | 48.97 | 27.48 | 22.83 | 24.82 | 24.63 |
| Arithmetic (mm) | SKEWNESS | 2.293 | 8.002 | 2.513 | 2.794 | 3.219 | 3.552 |
|  | KURTOSIS | 8.470 | 82.58 | 9.489 | 11.80 | 15.48 | 17.79 |
| METHOD OF | MEAN | 9.223 | 8.976 | 8.907 | 8.401 | 9.063 | 7.728 |
| MOMENTS | SORTING | 3.279 | 3.381 | 3.443 | 3.227 | 3.168 | 3.218 |
| Geometric (mm) | SKEWNESS | 0.036 | 0.307 | 0.212 | 0.121 | 0.097 | 0.241 |
|  | KURTOSIS | 2.420 | 3.084 | 2.480 | 2.552 | 2.658 | 2.724 |
| METHOD OF | MEAN | 6.761 | 6.800 | 6.811 | 6.895 | 6.786 | 7.016 |
| MOMENTS | SORTING | 1.713 | 1.757 | 1.783 | 1.690 | 1.664 | 1.686 |
| Logarithmic (f) | SKEWNESS | -0.036 | -0.307 | -0.212 | -0.121 | -0.097 | -0.241 |
|  | KURTOSIS | 2.420 | 3.084 | 2.480 | 2.552 | 2.658 | 2.724 |
| FOLK AND | MEAN | 9.314 | 8.835 | 8.891 | 8.336 | 8.997 | 7.600 |
| WARD METHOD | SORTING | 3.436 | 3.396 | 3.609 | 3.350 | 3.248 | 3.290 |
| (mm) | SKEWNESS | 0.044 | 0.061 | 0.099 | 0.050 | 0.028 | 0.063 |
|  | KURTOSIS | 0.989 | 1.014 | 1.037 | 1.024 | 1.019 | 1.016 |
| FOLK AND | MEAN | 6.746 | 6.823 | 6.813 | 6.906 | 6.796 | 7.040 |
| WARD METHOD | SORTING | 1.781 | 1.764 | 1.852 | 1.744 | 1.699 | 1.718 |
| (f) | SKEWNESS | -0.044 | -0.061 | -0.099 | -0.050 | -0.028 | -0.063 |
|  | KURTOSIS | 0.989 | 1.014 | 1.037 | 1.024 | 1.019 | 1.016 |
| FOLK AND | MEAN: | Medium Silt | Medium Silt | Medium Silt | Medium Silt | Medium Silt | Fine Silt |
| WARD METHOD | SORTING: | Poorly Sorted | Poorly Sorted | Poorly Sorted | Poorly Sorted | Poorly Sorted | Poorly Sorted |
| (Description) | SKEWNESS: | Symmetrical | Symmetrical | Symmetrical | Symmetrical | Symmetrical | Symmetrical |
|  | KURTOSIS: | Mesokurtic | Mesokurtic | Mesokurtic | Mesokurtic | Mesokurtic | Mesokurtic |
|  | MODE 1 (mm): | 6.710 | 6.710 | 5.980 | 5.980 | 6.710 | 5.980 |
|  | MODE 2 (mm): | 1.060 |  | 75.30 | 1.060 | 1.060 | 106.0 |
|  | MODE 3 (mm): |  |  |  |  |  |  |
|  | MODE 1 (f): | 7.222 | 7.222 | 7.388 | 7.388 | 7.222 | 7.388 |
|  | MODE 2 (f): | 9.884 |  | 3.734 | 9.884 | 9.884 | 3.240 |
|  | MODE 3 (f): |  |  |  |  |  |  |
|  | D10 (mm): | 1.967 | 1.933 | 1.860 | 1.848 | 2.043 | 1.732 |
|  | D50 (mm): | 8.860 | 8.386 | 8.177 | 8.029 | 8.801 | 7.272 |
|  | D90 (mm): | 48.49 | 44.40 | 56.19 | 41.00 | 40.38 | 35.61 |
|  | (D90 / D10) (mm): | 24.66 | 22.97 | 30.21 | 22.19 | 19.76 | 20.56 |
|  | (D90 - D10) (mm): | 46.52 | 42.46 | 54.33 | 39.16 | 38.34 | 33.87 |
|  | (D75 / D25) (mm): | 5.387 | 5.200 | 5.396 | 5.081 | 4.910 | 4.978 |
|  | (D75 - D25) (mm): | 17.50 | 16.25 | 16.25 | 15.07 | 15.96 | 13.52 |
|  | D10 (f): | 4.366 | 4.493 | 4.154 | 4.608 | 4.630 | 4.812 |
|  | D50 (f): | 6.818 | 6.898 | 6.934 | 6.961 | 6.828 | 7.103 |
|  | D90 (f): | 8.990 | 9.015 | 9.071 | 9.080 | 8.935 | 9.174 |
|  | (D90 / D10) (f): | 2.059 | 2.006 | 2.184 | 1.970 | 1.930 | 1.906 |
|  | (D90 - D10) (f): | 4.624 | 4.522 | 4.917 | 4.472 | 4.305 | 4.362 |
|  | (D75 / D25) (f): | 1.439 | 1.422 | 1.431 | 1.409 | 1.407 | 1.393 |
|  | (D75 - D25) (f): | 2.430 | 2.378 | 2.432 | 2.345 | 2.296 | 2.316 |
|  | % GRAVEL: | 0.0% | 0.0% | 0.0% | 0.0% | 0.0% | 0.0% |
|  | % SAND: | 6.7% | 6.3% | 8.8% | 5.8% | 5.5% | 4.9% |
|  | % MUD: | 93.3% | 93.7% | 91.2% | 94.2% | 94.5% | 95.1% |
|  | % V COARSE GRAVEL: | 0.0% | 0.0% | 0.0% | 0.0% | 0.0% | 0.0% |
|  | % COARSE GRAVEL: | 0.0% | 0.0% | 0.0% | 0.0% | 0.0% | 0.0% |
|  | % MEDIUM GRAVEL: | 0.0% | 0.0% | 0.0% | 0.0% | 0.0% | 0.0% |
|  | % FINE GRAVEL: | 0.0% | 0.0% | 0.0% | 0.0% | 0.0% | 0.0% |
|  | % V FINE GRAVEL: | 0.0% | 0.0% | 0.0% | 0.0% | 0.0% | 0.0% |
|  | % V COARSE SAND: | 0.0% | 0.0% | 0.0% | 0.0% | 0.0% | 0.0% |
|  | % COARSE SAND: | 0.0% | 0.3% | 0.0% | 0.0% | 0.0% | 0.0% |
|  | % MEDIUM SAND: | 0.0% | 0.7% | 0.0% | 0.0% | 0.0% | 0.0% |
|  | % FINE SAND: | 0.3% | 0.4% | 1.1% | 0.6% | 1.2% | 1.4% |
|  | % V FINE SAND: | 6.4% | 4.9% | 7.6% | 5.2% | 4.3% | 3.5% |
|  | % V COARSE SILT: | 10.3% | 9.1% | 8.0% | 8.1% | 8.9% | 7.1% |
|  | % COARSE SILT: | 16.3% | 16.0% | 14.1% | 16.0% | 17.6% | 15.0% |
|  | % MEDIUM SILT: | 20.8% | 20.8% | 20.5% | 21.0% | 22.0% | 20.6% |
|  | % FINE SILT: | 21.6% | 22.4% | 22.0% | 22.5% | 22.3% | 23.2% |
|  | % V FINE SILT: | 14.5% | 15.1% | 15.9% | 15.8% | 14.4% | 17.3% |
|  | % CLAY: | 9.9% | 10.1% | 10.7% | 10.8% | 9.4% | 11.9% |

**4) Grain size of bed material**

| Record Number | Sample Name | Measurement Date Time | Dx (10) | Dx (50) | Dx (90) | Skew [3] | Kurtosis [3] |
| --- | --- | --- | --- | --- | --- | --- | --- |
| 1 | IR4_Island_US | 12/03/2020 11:47 | 70.1 | 158 | 270 | 0.249 | -0.128 |
| 2 | IR4_Island_US | 12/03/2020 11:47 | 69.6 | 157 | 269 | 0.248 | -0.127 |
| 3 | IR4_Island_US | 12/03/2020 11:47 | 68.8 | 157 | 268 | 0.242 | -0.132 |
| 4 | Average of 'IR4_Island_US' | 12/03/2020 11:47 | 69.5 | 157 | 269 | 0.246 | -0.129 |
| 5 | IR2_b2g_US | 12/03/2020 12:05 | 105 | 201 | 330 | 0.168 | -0.122 |
| 6 | IR2_b2g_US | 12/03/2020 12:05 | 105 | 201 | 328 | 0.161 | -0.111 |
| 7 | IR2_b2g_US | 12/03/2020 12:06 | 105 | 201 | 329 | 0.168 | -0.113 |
| 8 | Average of 'IR2_b2g_US' | 12/03/2020 12:05 | 105 | 201 | 329 | 0.166 | -0.116 |
